# An overview of the data obtained during the validation of an optimized MALDI-TOF MS Biotyper database for the identification of anaerobic bacteria

**DOI:** 10.1016/j.dib.2018.04.070

**Published:** 2018-04-23

**Authors:** A.C.M. Veloo, H. Jean-Pierre, U.S. Justesen, T. Morris, E. Urban, I. Wybo, M. Kostrzewa, A.W. Friedrich, on behalf of the ENRIA workgroup

**Affiliations:** a University of Groningen, University Medical Center Groningen, Department of Medical Microbiology, Groningen, The Netherlands; b Centre Hospitalier Universitaire de Montpellier, Hôpital Arnaud de Villeneuve, Laboratoire de Bactériologie, 371 Avenue du Doyen Gaston Giraud, 34295 Montpellier Cedex 5, France; c Université Montpellier 1, UMR5119 ECOSYM, Equipe Pathogènes Hydriques Santé Environnements, UMR 5569 Hydrosciences, UFR Pharmacie, 15 avenue Charles Flahault, 34093 Montpellier Cedex 5, France; d Department of Clinical Microbiology, Odense University Hospital, Odense, Denmark; e UK Anaerobe Reference Unit, Public Health Wales Microbiology, Cardiff, UK; f Institute of Clinical Microbiology, University of Szeged, Hungary; g Department of Microbiology and Infection Control, Universitair Ziekenhuis Brussel, Brussels, Belgium; h Bruker Daltonics, Bremen, Germany

## Abstract

This data in brief article presents the data obtained during the validation of the optimized Biotyper Matrix Assisted Laser Desorption Ionization Time-of-Flight Mass Spectrometry (MALDI-TOF MS) database. The validation was performed by the different expertise laboratories, collaborating within the European Network for the Rapid Identification of Anaerobes (ENRIA) project, using 6309 human clinical anaerobic bacterial strains.

Different databases were compared with each other; the db 5989 database (V5 database); the V5 database complimented with Main Spectral Profiles (MSPs) of ENRIA strains added to the next update of the database; and the V5 database complimented with the MSPs of all anaerobic clinical isolates collected within the ENRIA project. For a comprehensive discussion of the full dataset, please see the research article that accompanies this data article (Veloo et al., 2018) [1]

**Specifications table**

**Table:** 

Subject area	*Medical Microbiology*
More specific subject area	*MALDI-TOF MS*
Type of data	*Table*
How data was acquired	*Biotyper, Matrix Assisted Laser Desorption Ionization Time-of-Flight Mass Spectrometry (Bruker Daltonics, Bremen, Germany)*
Data format	*Analyzed*
Experimental factors	*Assessment of the effect of the optimization of the Biotyper database for the identification of anaerobic bacteria was, by comparing the optimized database with the ‘old’ database.*
Experimental features	*Colonies of bacterial strains directly spotted on to a MALDI-TOF MS target plate and covered with matrix. If necessary, an on target extraction with 70% formic acid was performed prior to the addition of the matrix.*
Data source location	*Groningen, The Netherlands*
Data accessibility	*Provided with this article*

**Value of the data**

• Demonstrates how the Biotyper MALDI-TOF MS system performs for the identification of anaerobic genera commonly encountered in human clinical specimens.
• Highlights the performance of the Biotyper MALDI-TOF MS system with less commonly encountered genera/species of anaerobic bacteria (as it included a large number of isolates)
• Collaboration of specialist expertise laboratories yielded a MALDI-TOF MS database optimized for the identification of a significant number of anaerobic species.

## 1 Data

The data presented shows the performance of the system for the identification of anaerobic bacteria, prior to and after optimization of the database [1]. The obtained identification of each strain is categorized by genus. The log-score is used to assess the reliability of the identification. An increase in the log score was interpreted as a more reliable identification. Therefore the number of strains with a higher log score after optimization are also shown in Table 1.

**Table 1:** The MALDI-TOF MS data obtained during the validation of the for anaerobic bacteria optimized MALDI-TOF MS Biotyper database.

Strains (6309)	V5 database			V5 database+ENRIA (confirmed)			Higher score	Old database+ENRIA (all MSPs)		Higher score
	≤1.7	1.7–2	≥2	≤1.7	1.7–2	≥2		1.7–2	≥2	
										
*Acidaminococcus* spp. (7)										
* A. intestini* (7)		1	6		1	6	5	1	6	5
										
*Alistipes* spp. (8)										
* A. finegoldii* (4)			4			4			4	1
* A. onderdonkii* (3)			3			3			3	3
* A. indistinctus* (1)	1					1	1		1	1
										
*Alloscardovia* spp. (16)										
* A. omnicolens* (16)		2	14		2	14		2	14	
										
*Atopobium* spp. (58)										
* A. minutum* (6)			6			6	3		6	3
* A. parvulum* (25)		7	18		7	18		7	18	
* A. rimae* (15)	1	2	12	1	2	12	8	1	14	11
* A. vaginae* (4)			4			4	1		4	3
‘*A. detroitii*’ (3)	3			3					3	3
* Atopobium* spp. (5)	5			5					5	5
										
*Alloprevotella* spp. (1)										
* A. tannerae* (1)	1			1				1		1
										
*Bifidobacterium* spp. (52)										
* B. bifidum* (3)			3			3			3	
* B. breve* (15)		1	14		1	14	5	1	14	5
* B. catenulatum* (1)			1			1	1		1	1
* B. dentium* (13)		2	11		2	11		2	11	
* B. longum* (16)		9	7		9	7		9	7	
* B. scardovii* (4)			4			4	4		4	4
										
*Bilophila wadsworthia* (24)	7	15	2	2	5	17	20	7	17	22
										
*Bulleidia extructa* (3)			3			3			3	
										
*Butyricimonas* spp. (1)										
* B. virosa* (1)		1			1			1		
										
*Collinsella* spp. (4)										
* C. aerofaciens* (4)			4			4			4	
										
*Campylobacter* spp. (48)										
* C. concisus* (4)		1	3		1	3		1	3	
* C. fetus* (1)			1			1			1	
* C. rectus* (5)	2	1	2		2	3	5	2	3	5
* C. showae* (1)		1			1			1		
* C. hominis* (1)		1			1			1		
* C. ureolyticus* (34)		12	22		12	22		12	22	
* C. gracilis* (2)	2			2				1	1	2
										
*Cetobacterium* spp. (1)										
* C. somerae* (1)	1				1		1	1		1
										
*Desulfovibrio* spp. (6)										
* D. desulfuricans* (4)	4			1	1	2	3	2	2	4
‘*D. fairfieldenis*’ (2)	2			2					2	2
										
*Dialister* spp. (69)										
* D. micraerophilus* (21)		2	19			21	21		21	21
* D. pneumosintes* (48)		5	43		4	44	39	4	44	39
										
*Dielma fastidiosa* (2)	2			2					2	2
										
*Eubacterium* spp. (8)										
* E. brachy* (3)		1	2		1	2		1	2	
* E. limosum* (3)			3			3			3	
* Eubacterium* spp. (1)			1			1			1	
* E. tenue* (1)			1			1			1	
										
*Eggerthella lenta* (65)		10	55		10	55		10	55	
										
*Eggerthia catenaformis* (7)			7			7	3		7	5
										
*Flavonifractor plautii* (9)		1	8			9	6		9	6
										
*Helcococcus* spp. (15)										
*H. kunzii* (15)			15			15	2		15	2
										
*Lachnoanaerobaculum* spp. (9)										
* L. orale* (7)		2	5		2	5			7	3
* L. umeaense* (2)		2			2				2	2
										
*Leptotrichia* spp. (3)^a^			3			3			3	
										
*Megasphaera* spp. (1)										
* M. micronuciformis* (1)			1			1			1	
										
*Moryella indoligenes* (2)	2				1	1	2	1	1	2
										
*Mogibacterium* spp. (7)										
* M. timidum* (7)	7			7				6	1	7
										
*Filifactor* spp. (9)										
* F. alocis* (9)	9				1	8	9	1	8	9
										
‘Fenollaria massiliensis’ (7)	7			7					7	7
										
*Odoribacter* spp. (7)										
* O. splanchnicus* (7)	7			4		3	3		7	7
										
*Olsenella* spp. (7)										
* O. uli* (6)		1	5			6	5		6	5
* Olsenella* spp. (1)	1			1				1		1
										
*Ruminococcus* spp. (4)										
* R. gnavus* (4)		1	3			4	1		4	1
										
*Selenomonas* spp. (2)										
* S. artemidis* (2)	2					2	2		2	2
										
*Slackia* spp. (31)										
* S. exigua* (31)			31			31			31	
										
*Solobacterium moorei* (41)		4	37		1	40	32	1	40	32
										
*Sutterella* spp. (4)										
* S. wadsworthensis* (4)			4			4			4	
										
*Tissierella* spp. (1)										
* T. praeacuta* (1)			1			1			1	
										
*Actinomyces* spp. (306)										
* A. europaeus* (11)	2	6	3		2	9	10	2	9	10
* A. funkei* (3)		2	1		2	1		2	1	
* A. graeventizii* (20)		3	17		1	19	11	1	19	12
* A. israelii* (2)		2			2			2		
* A. meyeri* (5)		1	4		1	4		1	4	
* A. naeslundii* (7)		5	2		5	2		5	2	
* A. neuii* (37)		5	32		5	32		5	32	15
* A. odontolyticus* (121)		49	72		49	72		49	72	
* A. oris* (36)		7	29		7	29		7	29	
* A. radingae* (10)		4	6		3	7	8	3	7	8
* A. turicensis* (41)	2	10	29		10	31	28	10	31	28
* A. urogenitalis* (13)		2	11		2	11	2	2	11	5
										
*Veillonella* spp. (241)										
* V. atypica* (69)		3	66		3	66	1	1	68	46
* V. montpellierensis* (7)			7			7			7	
* V. ratti* (25)	2	17	6	2	17	6		3	22	22
* Veillonella* spp. (140)^b^		140			140			140		
										
*Blautia* spp. (1)										
* B. coccoides (1)*			1			1			1	
										
*Bacteroides* spp. (934)										
* B. caccae* (16)		1	15			16	5		16	5
* B. cellulosilyticus* (10)		1	9		1	9	2		10	6
* B. clarus* (2)		2				2	2		2	2
* B. coagulans* (11)	1	7	3	1	7	3	1	3	8	8
* B. eggerthii* (1)		1				1	1		1	1
* B. finegoldii* (2)			2			2			2	
* B. fragilis* (504)		5	499		5	499	81	5	499	81
* B. intestinalis* (2)		1	1			2	2		2	2
* B. massiliensis* (3)	2	1				3	3		3	3
* B. ovatus/xylanisolvens* (85)	2	16	67	2	16	67		10	75	68
* B. plebeius* (1)		1			1			1		
* B. pyogenes* (8)			8			8	1		8	1
* B. salyersiae* (10)			10			10	7		10	7
* B. thetaiotaomicron/faecis* (140)		4	136		3	137	10	3	137	48
* B. uniformis* (38)		1	37		1	37	3	1	37	3
* B. vulgatus/dorei* (91)		2	89		2	89		1	90	55
* B. nordii* (5)		2	3			5	3		5	3
* B. stercoris* (5)	1		4	1		4	2		5	3
										
*Clostridium* spp. (225)										
* C. aldenense* (5)			5			5	1		5	5
* C. baratii* (4)			4			4			4	
* C. bolteae* (1)			1			1	1		1	1
* C. butyricum* (11)			11			11			11	4
* C. cadaveris* (1)			1			1	1		1	1
* C. citronae* (7)		3	4		2	5	4	2	5	4
* C. clostridioforme* (23)		1	22		1	22	7	1	22	8
* C. colicanis* (1)		1			1			1		
* C. indolis* (3)			3			3			3	
* C. innocuum* (25)		12	13		12	13		12	13	
* C. paraputrificum* (7)			7			7			7	
* C. perfringens* (65)		5	60		5	60		4	61	2
* C. ramosum* (35)		3	32		3	32		3	32	
* C. sardiniense* (1)			1			1			1	
* C. scindens* (1)			1			1			1	
* C. septicum* (2)			2			2			2	
* C. sphenoides* (6)			6			6			6	
* C. sporogenes* (7)			7			7			7	
* C. symbiosum* (6)		2	4			6	6		6	6
* C. tertium* (10)		2	8		2	8		2	8	
* C. celatum* (2)			2			2			2	2
* Clostridium* spp. (2)		2			2			2		
										
*Paraclostridium* spp. (5)										
* P. bifermentans* (5)		4	1		4	1		4	1	
										
*Clostridioides* spp. (413)										
* C. difficile* (413)		17	396		17	396		17	396	
										
*Hungatella* spp. (16)										
*H. hathewayi* (16)			16			16			16	5
										
*Terrisporobacter* spp. (2)										
* T. glycolicus* (2)			2			2			2	1
										
*Paeniclostridium* spp. (10)										
* P. sordellii* (10)		1	9		1	9		1	9	3
										
*Intestinibacter* spp. (1)										
* I. bartletii* (1)		1			1			1		
										
*Hathewaya* spp. (2)										
* H. histolytica* (2)			2			2			2	
										
*Parabacteroides* spp. (54)										
* P. distasonis* (45)		1	44		1	44	24	1	44	24
* P. goldsteinii* (3)			3			3	3		3	3
* P. johnsonii* (1)			1			1	1		1	1
* P. merdae* (5)	1	4				5	4		5	5
										
*Prevotella* spp. (582)										
* P. amnii* (2)			2			2			2	2
* P. baroniae* (18)	1	1	16		2	16	13	2	16	13
* P. bergensis* (22)		3	19		2	20	17	2	20	17
* P. bivia* (112)		8	104		8	104		5	107	88
* P. buccae* (64)		5	59		5	59		5	59	2
* P. buccalis* (15)	7	7	1		4	11	14	4	11	14
* P. copri* (2)			2			2			2	
* P. corporis* (14)		3	11		1	13	9		14	12
* P. dentalis* (5)			5			5			5	4
* P. denticola* (39)			39			39	22		39	22
* P. disiens* (25)		3	22		3	22	2	1	24	6
* P. histicola* (9)		1	8		1	8	5	1	8	5
* P. intermedia* (27)	1	5	21	1	4	22	6	4	23	22
* P. jejuni* (5)	4	1		4	1				5	5
* P. loescheii* (1)		1			1				1	1
* P. maculosa* (2)			2			2			2	
‘*P. massiliensis*’ (2)	2			2					2	2
* P. melaninogenica* (64)	5	15	44	5	15	44		14	50	48
* P. heparinolytica* (13)			13			13	7		13	7
* P. nanceiensis* (14)		2	12		2	12	10	2	12	10
* P. nigrescens* (48)	1	7	40	1	7	40	10	6	42	39
* P. oris* (13)			13			13	4		13	4
* P. pallens* (1)			1			1			1	
* P. oulorum* (3)		1	2		1	2	2	1	2	2
* P. salivae* (11)	6	5				11	11		11	11
* P. timonensis* (42)	2	9	31	1	1	40	38	1	41	40
* P. veroralis* (2)		1	1			2	2		2	2
* P. oralis* (3)	1	2				3	3		3	3
* P. veroralis* (1)		1				1	1		1	1
*Prevotella* spp. (3)			3			3			3	3
										
*Fusobacterium* spp. (303)										
* F. canifelinum* (1)		1			1			1		
* F. gonidiaformans* (16)			16			16	4		16	4
* F. necrophorum* (52)		2	50		1	51	16	1	51	18
* F. nucleatum* (200)	6	60	134	6	60	134		47	153	82
* F. periodonticum* (14)		13	1		13	1		13	1	
* F. ulcerans* (5)			5			5	2		5	2
* F. varium* (3)			3			3			3	1
* Fusobacterium* spp. (12)	1	4	7	1	4	7		5	7	1
										
*Anaerococcus* spp. (230)										
* A. hydrogenalis* (12)		4	8		4	8		4	8	
* A. lactolyticus* (11)		5	6		1	10	10	1	10	10
* A. murdochii* (34)	2	4	28	1	4	29	18	4	30	24
* A. degeneri* (8)	5	3		5	3			1	7	8
* A. octavius* (6)	1		5	1		5			6	1
* A. prevotii* (3)		2	1		2	1		2	1	
* A. tetradius* (7)		5	2		5	2		5	2	
* A. vaginalis* (107)	30	64	13	11	37	59	55	16	91	107
* Anaerococcus* spp. (28)	1	4	23	1	4	23		5	23	1
* A. senegalensis* (10)	9	1		9	1				10	10
* A. nagyae* (4)	4			4				1	3	4
										
*Finegoldia magna* (412)		87	325		87	325		87	325	
										
*Murdochiella asaccharolytica* (13)		5	8		4	9	6	4	9	6
										
*Peptoniphilus* spp. (349)										
* P. duerdenii* (7)	7			7					7	7
* P. olsenii* (8)	8					8	8		8	8
* P. tyrrelliae* (4)	4					4	4		4	4
* P. rhinitidis* (8)	8			8					8	8
* P. koenoeneniae* (1)	1					1	1		1	1
* P. lacrimalis* (20)	20			1	1	18	19	1	19	20
* P. gorbachii* (12)	1	1	10	1		11	10	1	11	12
‘P. grossensis’ (18)	13	5		13	5				18	18
* P. harei* (241)	4	41	196	2	39	200	26	20	221	192
* P. ivorii* (1)			1			1			1	1
* P. coxii* (27)		10	17		5	22	17		27	27
* P. asaccharolyticus* (2)		2			2			2		
										
*Peptostreptococcus* spp. (130)										
* P. anaerobius* (98)		7	91		4	94	73	4	94	73
* P. stomatis* (32)	31	1		31	1			8	24	32
										
*Peptococcus niger* (7)	1	6			2	5	7	2	5	7
										
*Parvimonas micra* (244)		20	224		20	224		20	224	
										
*Porphyromonas* spp. (129)										
* P. asaccharolytica/uenonis* (33)	27	4	2	27	4	2		11	22	27
* P. gingivalis* (7)			7			7			7	
* P. somerae* (75)	3	23	49	3	14	58	47	15	60	50
* Porphyromonas* spp. (1)			1			1			1	
* P. macacae* (2)			2			2	2		2	2
* P. bennonis* (11)	6	2	3	6	2	3		2	9	10
										
*Cutibacterium* spp. (647)										
* C. acnes* (556)		86	470		75	481	285	75	481	285
* C. avidum* (72)		25	47		25	47		21	51	12
* C. granulosum* (19)		7	12		7	12	2	5	14	7
										
*Propionibacterium* spp. (26)										
* P. freundenreichii* (1)		1			1			1		
* Propionibacterium* spp. (25)		5	20		5	20		5	20	
										
*Propionimicrobium lymphophilum* (30)		28	2		28	2		28	2	
										
No. ID (458)	458			458						
Totals (*n*)	760	1064	4485	654	937	4718	1205	852	4999	2219
%	12.0%	16.9%	71.1%	10.4%	14.9%	74.8%	19.1%	13.5%	79.2%	35.2%

a All three strains were only identified at the genus level with a log score ≥2.

b These strains also included the species *V. dispar*, *V. parvula*, *V. denticariosi* and *V. rogosae*.

## 2 Experimental design, materials and methods

### 2.1 Bacterial strains

The expertise laboratories:

University Medical Center Groningen (UMCG), Groningen, The Netherlands;
Centre Hospitalier Universitaire de Montpellier, Montpellier, France;
Odense University Hospital, Odense, Denmark;
UK Anaerobe Reference Unit (UKARU), Public Health Wales Microbiology, Cardiff, United Kingdom; University of Szeged, Szeged, Hungary and
Universitair Ziekenhuis Brussel, Brussels, Belgium.

All utilized 6 months' worth of anaerobic human clinical isolates encountered and identified using the MALDI-TOF MS Biotyper system (Bruker Daltonics, Bremen, Germany), which resulted in a total of 6309 isolates used for validation. The obtained spectra were compared with the V5 database, the V5 database plus the ENRIA MSPs which were added to the next update of the database and the V5 database plus all MSPs created from the collected ENRIA strains. All MSPs were created and supplied by Bruker Daltonics.

### 2.2 Identification

The MALDI-TOF MS measurements were performed at each laboratory as described previously [2]. The measurements were performed as part of the daily routine, using standard settings. Obtained log scores were interpreted as advised by the manufacturer.

### 2.3 Data interpretation

The identifications obtained were divided into 3 groups.

Group 1 (log score <1.7)=reliable identification.

Group 2 (log score ≥1.7 and <2)=identification with low confidence e.g. reliable genus only.

Group 3 (log score ≥2)=identification with high confidence e.g. reliable species.

Identifications to the subspecies level were not considered during the data analyses.

Species that cannot be differentiated from each other using MALDI-TOF MS were presented as such: e.g. *Bacteroides ovatus/xylanisolvens, Bacteroides thetaiotaomicron/faecis*, *Bacteroides vulgatus/dorei* and *Fusobacterium nucleatum/naviforme*.

Species that cannot be reliably identified at the species level using 16S rRNA sequencing were assumed to be either: e.g. *Porphyromonas asaccharolytica*/*uenonis*. This included strains identified as *Veillonella dispar*, *Veillonella parvula*, *Veillonella denticariosi and Veillonella rogosae*. These strains were categorized as being *Veillonella* species, regardless of the obtained log score. No differentiation was made between valid and non-valid species.
